# Low-Power Ternary Bipolar Memristor of Naturally Oxidized Porous Ti_3_C_2_T_*x*_ MXene Flakes

**DOI:** 10.1021/acsomega.5c02640

**Published:** 2025-06-19

**Authors:** Seyed Mehdi Sattari-Esfahlan, Ali Shayesteh Zeraati, Jae-Hyun Lee, Uttandaraman Sundararaj, Reza Rahighi

**Affiliations:** † 27259 Institute for Microelectronics (TU Wien), Gusshausstrasse 27-29, Vienna 1040, Austria; ‡ Department of Material Science and Engineering, Ajou University, Suwon 16499, Korea; § Department of Chemical and Petroleum Engineering, University of Calgary, 2500 University Dr NW, Calgary T2N 1N4, Canada; ∥ Department of Mechanical and Industrial Engineering, University of Toronto, Toronto, Ontario M5S 3G8, Canada; ⊥ Department of Electrical and Computer Engineering, Sungkyunkwan University, Suwon 16419, Republic of Korea; # Institute of Materials Science and Nanotechnology, 52948 Bilkent University UNAM, Ankara 06800, Türkiye

## Abstract

The multilevel characteristic of a single memristor cell offers a promising alternative to using multiple FETs for the same data storage capacity. Ti_3_C_2_T_*x*_ MXene, with its atomically thin structure and tunable surface-dependent electronic properties, is a strong candidate for multifunctional electronic materials and devices. However, the high conductivity of as-prepared Ti_3_C_2_T_*x*_ flakes limits their use in active electronic devices. Here, we present naturally oxidized porous Ti_3_C_2_T_*x*_ MXene flakes as promising materials for ion-based resistive switching memory (RSM) applications. The fabricated devices exhibit reproducible ternary memory behavior with low operating voltage, stable retention, and robust performance. We suggest that the drift of oxygen ions and the formation of metallic filaments in oxidized porous Ti_3_C_2_T_*x*_ are responsible for observed resistive switching. Our findings demonstrate that oxidized porous Ti_3_C_2_T_*x*_ MXene can be an alternative to traditional resistive switching materials and enhance multilevel memory technology.

## Graphical abstract

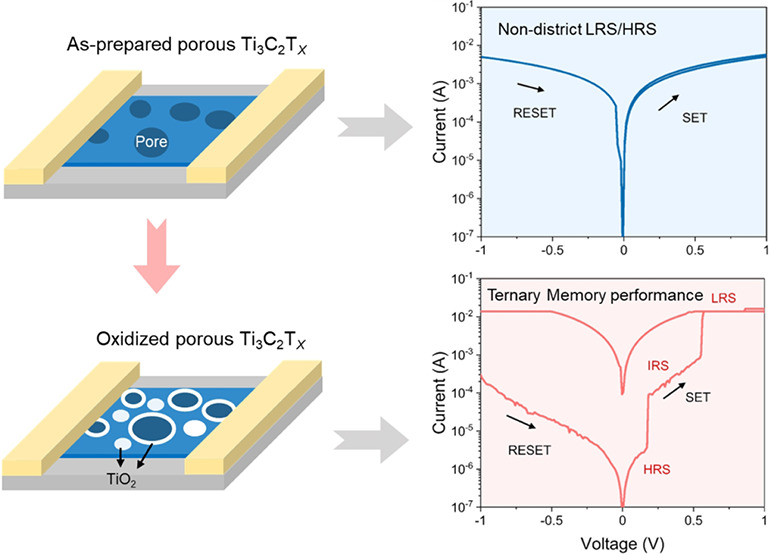

## 1 Introduction

Memristors have demonstrated significant potential for applications in data storage,^1^ logic operations,^2^ and neuromorphic computing^3^ due to their low operating voltages, high resistance ON/OFF ratios, and long retention times. These devices function through resistive switching mechanisms involving ion migration^4^ and redox reactions,^5^ enabling transitions between high and low resistance states. However, challenges persist, such as complex phase transitions, instability caused by ion migration, and difficulties in precisely controlling resistive switching performance. Addressing these issues is crucial for improving the reliability and scalability of memristors in advanced electronic systems. A promising approach to enhancing the functionality of memory devices involves the modification of 2D materials like Ti_3_C_2_T_*x*_ MXenes. Oxidation of Ti_3_C_2_T_*x*_ MXene flakes, which converts −OH groups on the surface to —O groups, significantly alters the material’s band structure,^6^ allowing it to integrate into both semiconducting and insulating media for diverse electronic applications.^7,8^ Notably, Ti_3_C_2_T_*x*_ MXene is more promising than nitride MXenes as a semiconductor due to its high, nearly free-electron-state (NFES) density, which enhances conductivity within single layers and through stacked films. In MXenes such as Ti_3_C_2_T_*x*_, d-electrons and delocalized p-electrons contribute to high conductivity. Recent studies reveal NFES near the Fermi level, supporting metallic behavior.^9–11^ Surface terminations significantly affect electron density: −O terminations enhance metallicity, while −F and −OH reduce it. Replacing C with N alters the bonding and reduces delocalization, leading to lower conductivity. Thus, Ti_3_C_2_T_*x*_’s unique Ti–C bonding, NFES, and tunable surface chemistry make it a strong candidate for electronic and energy applications. However, surface adsorbents such as water and oxygen can induce p-doping, reducing the^12^ conductivity by acting as electron acceptors, as observed in recent studies.^13,14^ Irreversible oxidation at the edges and on the surface of Ti_3_C_2_T_*x*_ MXene flakes creates native MXene-oxide interfaces, enabling a semiconducting platform suitable for electronic^7,15^ and neuromorphic devices.^16,17^ This native oxide formation eliminates the need for seeding layers in oxide insulator growth on 2D materials,^18^ ensuring ultraclean interfaces with host layers.^19,20^ Recent research has demonstrated oxidized Ti_3_C_2_T_*x*_ MXene and its composites in nonvolatile resistive random-access memory (RRAM),^21^ ferroelectric nonvolatile memory,^22^ floating-gate transistor memory,^7^ and active switching layers in memory devices,^23–25^ which exhibit typical binary resistive switching behavior. Moreover, introducing porosity into MXene layers can reduce electrical conductivity by weakening electrostatic connections, a concept observed in porous graphene-based devices.^26–30^ While these advances in porous Ti_3_C_2_T_*x*_ MXene-based resistive switching memories (RSMs) are promising, the exploration of nonbinary resistive switching mechanisms in these materials remains underdeveloped and warrants further investigation.

Here, we report an RSM device utilizing naturally oxidized porous Ti_3_C_2_T_*x*_ MXene, which exhibits reproducible ternary switching performance. We demonstrate robust multibit switching with a high endurance of 2000 s, an extensive On/Off ratio, and three stable resistive states over 300 cycles, all with high uniformity. The number of intermediate states is primarily influenced by the thickness of the a-BN film, which governs the evolution of filament dimensions within the channel and drives multistate switching behavior. The ternary resistive switching demonstrated here has the potential to significantly enhance the data capacity of current binary memory technology based on 2D materials.

## 2 Experimental Procedure

### 2.1 Synthesis and Characterization of Oxidized Ti_3_C_2_T_*x*_MXene

The Ti_3_C_2_T_*x*_ MXene was synthesized using the evaporated-nitrogen minimally intensive layer delamination (EN-MILD) method^31^ (Figure 1a). In our EN-MILD synthesis approach, the etching reaction was carried out under a continuous dry nitrogen flow (∼250 SCCM), gradually concentrating the etching solution by partial evaporation. A mixture of 1.6 g LiF in 20 mL of 9 M HCl was stirred at 50 °C under nitrogen, and 1 g of Ti_3_AlC_2_ was added over 30 min. The reaction was conducted for various durations (30 h) to assess the effect of nitrogen purging on MXene formation. Postetching, the material was extensively washed with Millipore water by repeated centrifugation (∼800 mL total) until near-neutral pH. Delamination was performed via 10 min of ice-bath sonication under nitrogen. To isolate a few-layer Ti_3_C_2_T_*x*_ flakes, the suspension was centrifuged (1 h, 3500 rpm), suspended in DI water, stirred, and sonicated before being transferred to the substrate. The Ti_3_C_2_T_*x*_ film was oxidized by exposing it and drying under open-air conditions for a week. The high-angle annular dark-field scanning transmission electron microscopy (HAADF-STEM) image of the synthesized Ti_3_C_2_T_*x*_ MXene and the corresponding energy-dispersive X-ray spectroscopy (EDX) elemental mapping demonstrate the distributions of Ti, C, O, and F in the oxidized Ti_3_C_2_T_*x*_ (Figure 1b). X-ray photoemission spectroscopy (XPS) analysis of oxidized Ti_3_C_2_T_*x*_ MXenes shows four elemental peaks at binding energies of 284 eV (C 1s), 459 eV (Ti 2p), 530 eV (O 1s), and 685 eV (F 1s).^14^ An enlarged O 1s peak appeared around 532 eV, indicating the formation of TiO_2_ in the Ti_3_C_2_T_*x*_ film naturally oxidized in an air-conditioned environment (Figure 1c). In particular, hydrolysis-assisted annealing is initiated by the excessive −O and −OH groups,^32^ and such fluorinated and oxygenated sites on the oxidized Ti_3_C_2_T_*x*_ reduce electrical conductivity.^33^ Additionally, the X-ray diffraction (XRD) patterns reveal a (006) TiO_2_ peak that appears after oxidation of the sample (Figure 1d). We conducted high-resolution HAADF-STEM imaging of oxidized Ti_3_C_2_T_*x*_ to confirm the film morphology. As shown in Figure 1e, the formation of TiO_2_ clusters with a lattice spacing of ∼0.35 nm is observed.^34^ Notably, one can see a large number of pores with varied sizes, ranging from a few angstroms (created by single atomic vacancies) to larger pores with dimensions of a few nanometers. We believe that the pores are possibly generated during hydrofluoric acid (HF) etching^31^ and potentially act as trapping sites for water molecules and moisture.

**Figure 1:**
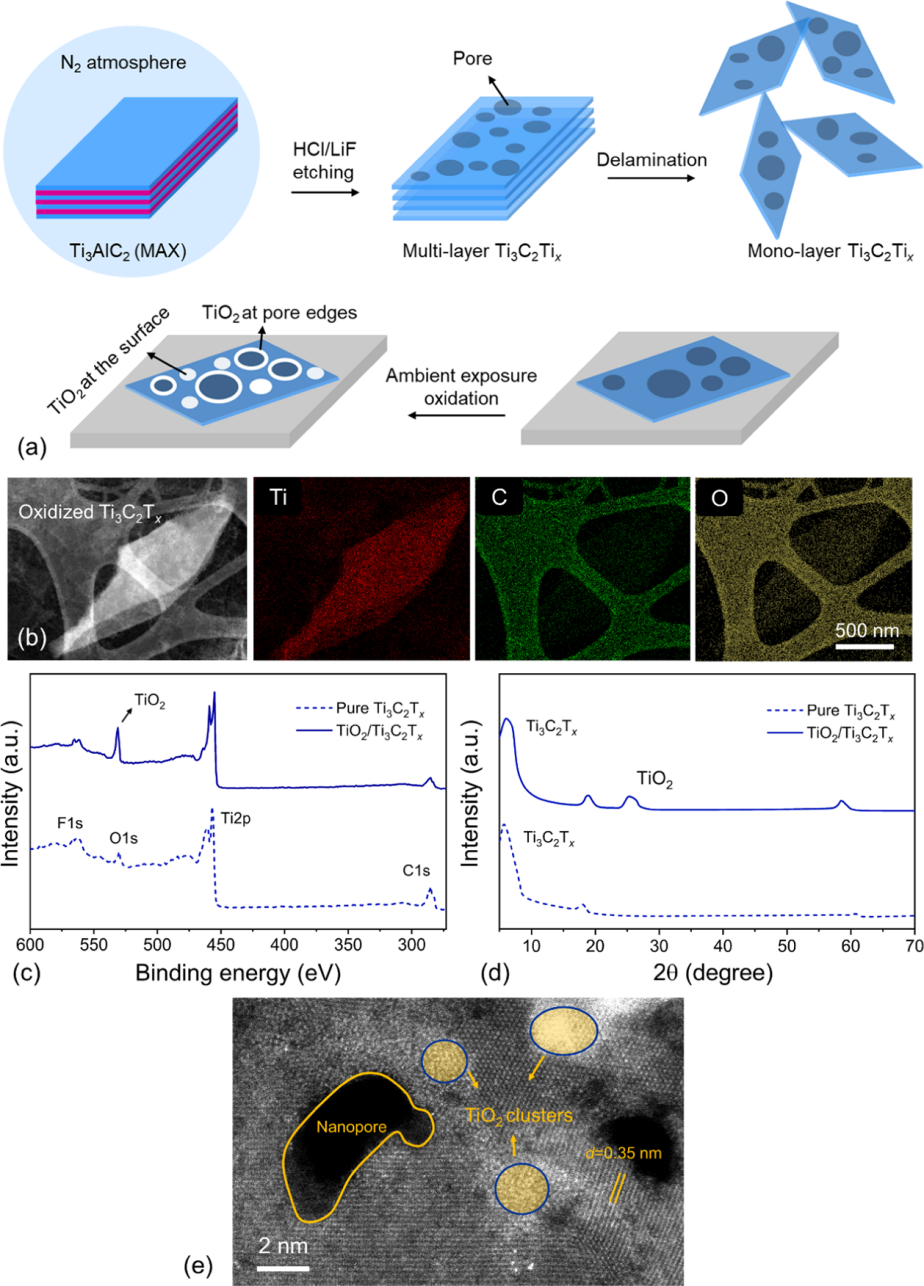
(a) Schematic of EN-MILD synthesis approach and ambient oxidation. (b) STEM and the corresponding EDS mapping of monolayer-oxidized Ti_3_C_2_T_*x*_ MXene (scale bar is 500 nm). (c) XPS and (d) XRD of pure and oxidized Ti_3_C_2_T_*x*_ MXene. (e) HAADF-STEM images of naturally oxidized Ti_3_C_2_T*x* MXene. The scale bar is 2 nm.

### 2.2 Device Fabrication

The oxidized Ti_3_C_2_T_*x*_ film was fabricated using the spin-coating approach on the SiO_2_ substrate. Then, the structure was fabricated by patterning the electrode area with electron beam lithography, followed by the deposition of Ag and Au electrode metals (50 nm) on the SiO_2_ substrate. Eventually, a metal lift-off was performed to remove excess metals and achieve the anode and cathode electrodes. Figure 2a shows a schematic illustration of our fabricated device.

**Figure 2:**
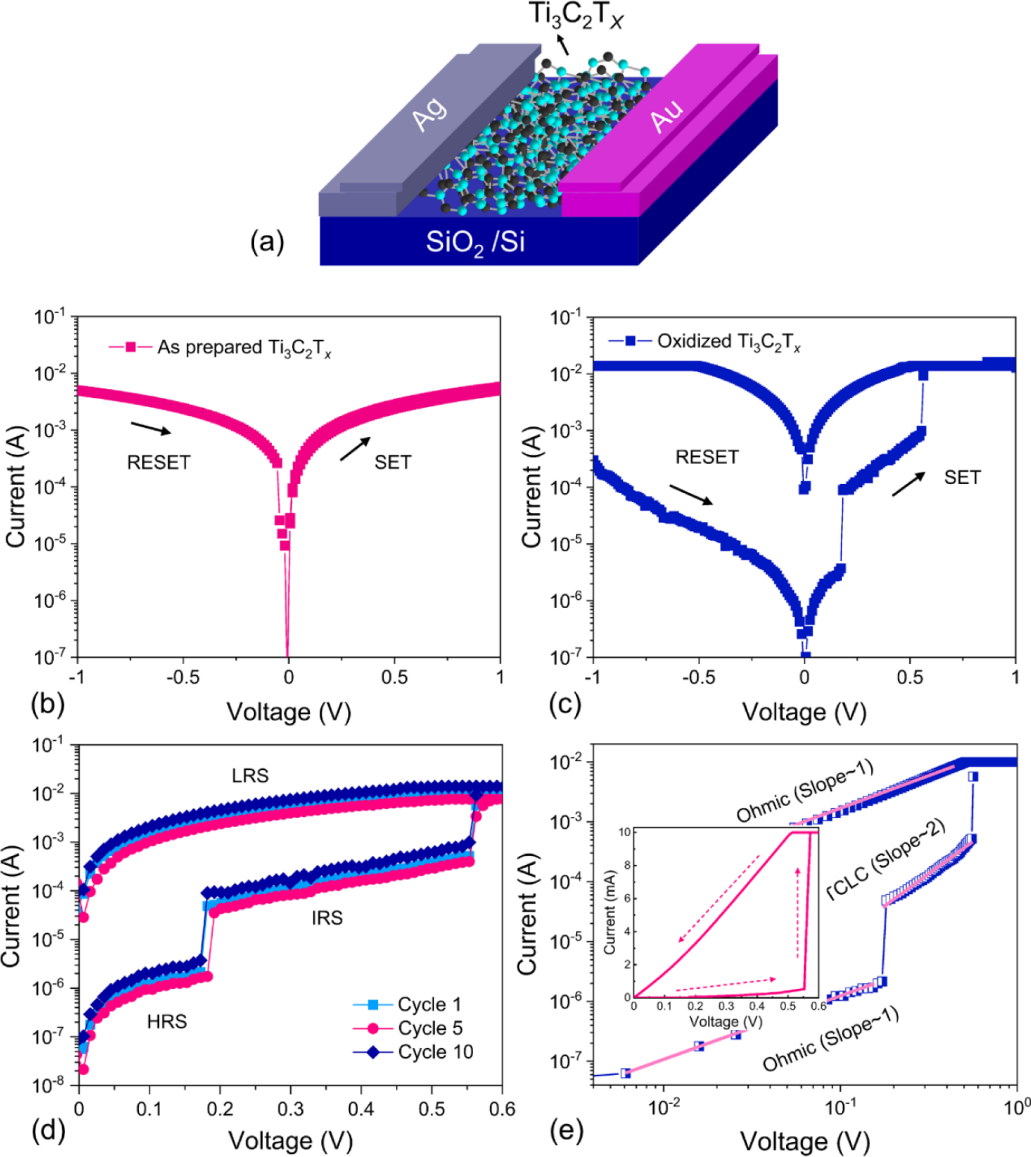
(a) Schematic and typical *I*–*V* characteristics of (b) as-prepared and (c) the porous Ti_3_C_2_T_*x*_ MXene RSM during SET and RESET. (d) Ternary resistive switching behavior of *I*–*V* characteristics with intermediate level states at positive voltages during ten consecutive cycles. (e) The double-log illustration and the full linear plot (inset) for the same data for physical transport analyses.

### 2.3 Electrical Characterization

The electrical characterization was performed on a Ag/oxidized-Ti_3_C_2_T_*x*_/Au device using a semiconductor characterization analyzer (Keithley 4200SCS) under ambient conditions and at room temperature.

## 3 Results and Discussion

The electrical performance of the device incorporating a pristine (as-prepared) porous MXene layer is presented in the current–voltage (*I*–*V*) characteristics shown in Figure 2b. The device exhibits negligible resistive switching behavior, with an almost imperceptible memory window and indistinct resistance states during the SET and RESET processes. In contrast, when the porous MXene layer undergoes controlled oxidation, the resulting device demonstrates clear bipolar resistive switching under identical measurement conditions. The oxidized porous MXene-based device displays a pronounced memory window with an On/Off current ratio exceeding 4 orders of magnitude, indicating a significant enhancement in switching behavior due to surface oxidation. Initially, the anode electrode (Ag) and the cathode electrode (Au) are electrostatically isolated by the high-resistance porous oxidized Ti_3_C_2_T_*x*_ film; thus, the device is in a high-resistance state (HRS). During the setting process, the device remains in HRS while sweeping the bias voltage from zero to ∼ 0.16 V. With further enhancement in bias voltage, an abrupt upswing in the current is observed at ∼0.59 V, forming an intermediate resistive switching state (IRS). Eventually, the device switches to a low-resistance state (LRS) after exhibiting multilevel RS. Three resistance states—“0” (HRS), “1” (IRS), and “2” (LRS)—can be regarded as ternary memory behavior. The current gap between the three resistance levels is high, ensuring a minimal possibility of common reading and writing errors. During the resetting process, the memory device exhibited a binary RS function with an On/Off current ratio of about 4 orders of magnitude, almost as large as in the SET process. As seen in Figure 2d, for 10 repetitive cycles, stable multilevel switching is observed. The current level between the three different RS states of LRS, IRS, and HRS is above 1 order of magnitude. The *I*–*V* characteristics with HRS, IRS, and LRS are replotted in double logarithmic coordinates, as shown in Figure 2e, and the switching area is divided into three regions with different slopes. The same *I*–*V* curve is illustrated on a decimal plot (inset) for transport analysis. We observed almost ohmic behavior during a forward sweep (HRS regime). The *I*–*V* curve showed a trap-charge-limited current (TCLC) in the IRS region,^30,35^ and the current over the reverse sweep was observed to be ohmic with excellent linearity (inset figure), offering a significant evolution in electrical transport behavior.

Memory function was examined over a high number of cycles to check the repeatability of the RS behavior. The retention quality of our Ti_3_C_2_T_*x*_ MXene RSM device is shown in Figure 3a. Our device maintains three discrete resistive levels over a duration of more than 2 × 10^3^ seconds at the reading voltage of *V*_READ_ = 0.05 V, effectively countering the common issue of leakage current in typical 2D switching channels. Additionally, the device demonstrated stable switching over hundreds of cycles with a robust resistance level gap between the three resistive states, showing the potential to minimize reading and writing errors in the device (Figure 3b). The voltage uniformity of each state is a key parameter in analyzing memory device reliability. Thus, we examined the uniformity of *V*_SET_, *V*_IRS_, and V_RESET_ for several devices, as shown in Figure 3c. *V*_SET_, *V*_SET_, and *V*_RESET_ are determined to be in the 0.19, 0.56, and 1.05 V voltage ranges, respectively. This reveals the significant reliability of the fabricated device while switching among the three different resistive switching levels.

**Figure 3:**
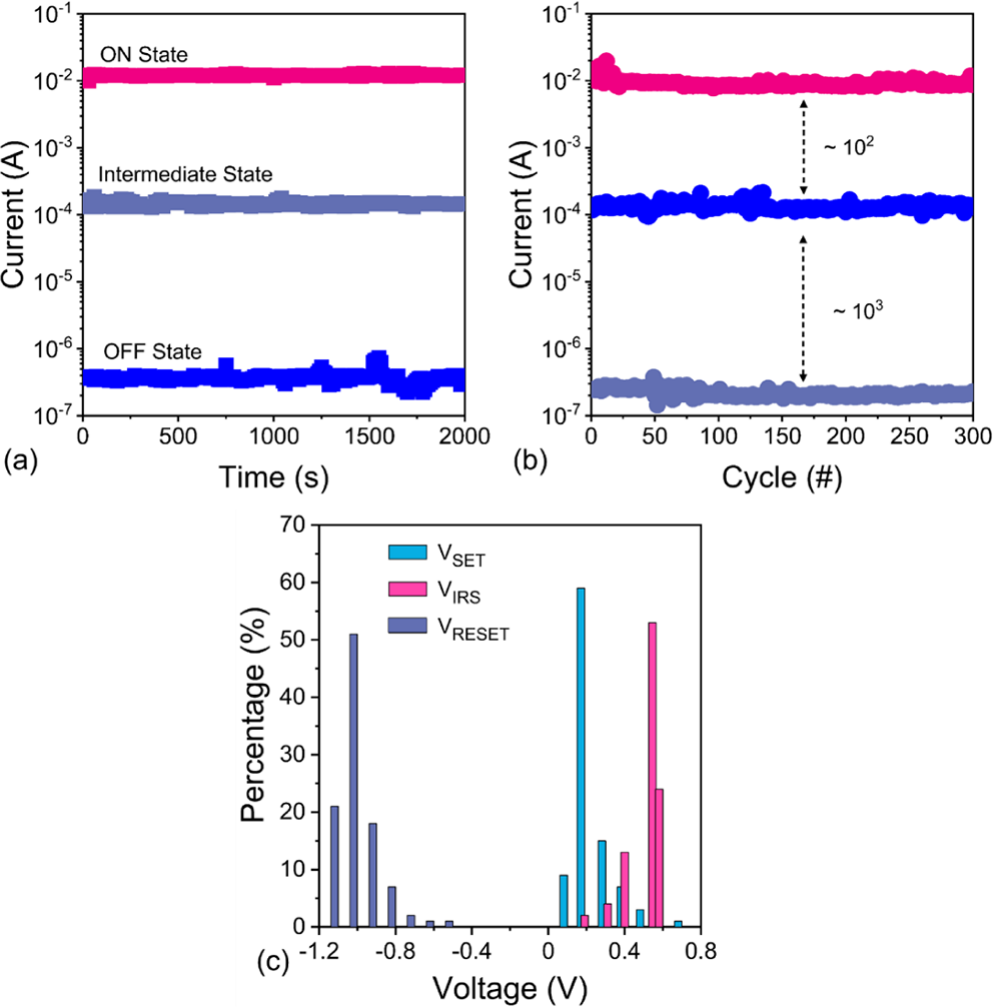
(a) Retention, (b) endurance performance with five different resistance levels, and (c) variations in *V*_SET_, *V*_IRS_, and *V*_RESET_ of the MXene RSMs at *V*_READ_ = 0.05 V.

The device’s resistive switching mechanism has been investigated in detail, as illustrated in Figure 4. Two main factors are more likely to cause ternary resistive switching behavior in oxidized porous Ti_3_C_2_T_*x*_ MXene: the channel’s structural impact and the role of the Ag electrode. In the former case, it is likely that most of the traps are located in pores and vacancies in the body of the Ti_3_C_2_T_*x*_ flakes. At low bias voltages, negatively charged oxygen ions are driven by the positive bias and form local ion clusters in the channel, which grow larger and overlap with further enhancement of the electric field. The drift of these ions toward the electrode causes an initial current path through the channel. Exclusively, segregated pores can lead to localized states that capture electrons and suppress the higher conductivity in the HRS, in which the *I*–*V* characteristic follows Ohm’s law. Moreover, the oxidized porous Ti_3_C_2_T_*x*_ can form an electrolyte-like ionic conducting platform and pave a path for Ag^+^ cations (existing due to the Ag electrode oxidation at the Ti_3_C_2_T_*x*_/electrode interface) to migrate through the channel. For instance, various functional groups −F and −O in particular have the potential to interact with metal ions such as Ag+ cations that can diffuse into the porous Ti_3_C_2_T_*x*_.^36^ As the bias crosses 0.19 V, the diffusion of Ag ions into the Ti_3_C_2_T_*x*_ layer forms the conducting filament. The rise of an IRS with a distinct bias is caused by the formation and alteration of the Ag filament dimension, altering channel resistance. Also, oxygen ions continue migrating toward the electrode, albeit with less intensity since most of them have already diffused at the early stage of bias voltage. By further increasing the bias voltage, the charge traps are filled, and the current follows TCLC conduction. When the bias exceeds 0.53 V, the device switches from IRS to the LRS. At higher biases, Ag ions have a further chance to diffuse, which enlarges the filament size (or number) and reaches the electrode, ultimately resulting in LRS in the device with an ohmic behavior. Note that the transition from HRS to LRS can still involve a weaker drift of oxygen ions in parallel with changes in Ag filament dimensions/numbers.^4^ During the negative bias application, the Ag filaments break down at the narrowest spot, possibly due to the generated Joule heating during LRS,^37^ resetting the memristor to HRS.

**Figure 4:**
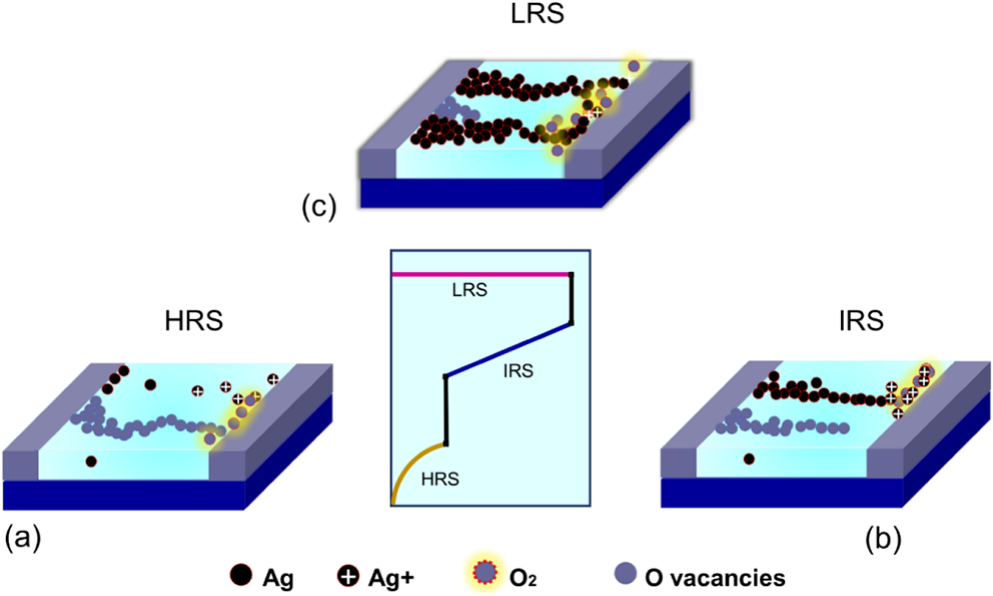
Mechanism of resistive switching in Ti_3_C_2_T_*x*_ MXene oxide RSM in (a) HRS, (b) IRS, and (c) LRS.

Exclusively, regarding TiO_2_ formation and its influence on resistive switching, it is important to emphasize that partial surface oxidation of Ti_3_C_2_T_*x*_ leads to the formation of TiO_2_ nanodomains (STEM image in Figure 1d), predominantly at defect sites or sheet edges. Under an applied electric field, oxygen vacancies within these TiO_2_ regions can migrate and accumulate, facilitating the formation and rupture of conductive filaments and thereby enabling the reversible SET/RESET behavior characteristic of our memory devices. Additionally, the natural oxidation process may introduce mobile oxygen vacancy sites, which act as effective nucleation centers for filament growth, altering both switching uniformity and device endurance. The bandgap contrast between TiO_2_ and pristine Ti_3_C_2_T_*x*_ forms a heterojunction within the switching layer, modulating the electrostatic potential and enabling improved charge trapping/detrapping dynamics. Such interface engineering results in more distinct resistance states and supports low-power operation through effective barrier control at the Ti_3_C_2_T_*x*_–TiO_2_ interfaces. In terms of chemical stability, TiO_2_ has the potential to provide a protective encapsulation effect, enhancing long-term data retention and cycling reliability of the device. Collectively, these features underscore the critical role of controlled TiO_2_ formation in optimizing MXene-based resistive memory devices.

Note that beyond a common binary resistive switching performance, the number and size of these conductive paths can be altered under different compliance currents (*I*_CC_), resulting in different switching levels in the device. However, in all devices, intermediate switching states appear without any modification in *I*_CC_, which is one of the features that make our device unique among other multilevel memories. Also, the migration and extrusion of Ag^+^ cations in oxidized porous Ti_3_C_2_T_*x*_ are analogous to the migration of Ca^2+^ ions in the biological synapse. Thus, our device can mimic biological processes, suggesting a novel ion-based memory platform for neuromorphic computing.

Table 1 summarizes key performance metrics of two-terminal resistive switching devices based on pristine and oxidized Ti_3_C_2_T_*x*_ layers. In general, our results show that pristine MXene devices exhibit weak memory behavior, characterized by low On/Off ratios and poor switching stability, likely due to insufficient defect density and uncontrolled filament formation. In contrast, our oxidation strategy targeting vacancy-induced TiO_2_ formation yields a hybrid TiO_2_–MXene structure that markedly enhances switching uniformity, endurance, and On/Off performance. These improvements underscore the effectiveness of controlled microoxidation in engineering MXene-based resistive memory.

**Table 1:** Two-Terminal Resistive Switching Devices Based on Ti_3_C_2_T_*x*_ MXene Oxide and Related Parameters

Device structure	On/Off ratio	Endurance	Retention	*V*_SET_	Switching levels	ref
Ag/Ti_3_C_2_T_*x*_/Pt	∼10	500	-	0.55 V	2 (Binary)	*Small 2024, 20, 2400165*
Al/Ti_3_C_2_T_*x*_/Pt	∼10^2^	-	-	5 V	2 (Binary)	*Small* 2019, 15, 1900107
Ag*/*Ti_3_C_2_T_*x*_/ITO	∼10^3^	10^3^	10^2^ s	7 V	2 (Binary	*ACS Appl. Mater. Interfaces 2024, 16, 17821–17831*
rGO/HT-Ti_3_C_2_T_*x*_/Pd	∼10^2^	-	-	12 V	2 (Binary	*npj 2D Mater. and Appl. (2023) 7*
rGO/Ti_3_C_2_T_*x*_-PVP/Au	∼10	∼40	8 × 10^3^ s	1 V	2 (Binary)	*ACS Appl. Mater. Interfaces 2019, 11, 38061–38067*
Mo_2_TiC_2_T_*x*_/FE-Ti_3_C_2_T_*x*_/Mo_2_TiC_2_T_*x*_	∼10^2^	200	10^4^ s	∼4 V	2 (Binary)	Appl. Phys. Lett. 2023, 123, 013503
Ag*/*Ti_3_C_2_T_*x*_-TiO_2_/Pt	∼10^6^	-	-	∼3.2 V	2 (Binary)	*Adv. Electron. Mater. 2021, 7, 2000866*
Au*/*Ti_3_C_2_T_*x*_-TiO_2_/Ag	∼10^2^	100	10^3^ s	∼2.5 V	2 (Binary)	*Adv. Mater. 2023, 35, 2303737*
Pt*/*Ti_3_C_2_T_*x*_-TiO_2_/ITO	∼10	-	10^4^ s	∼2 V	2 (Binary)	*Carbon* 2023, 205, 365–372
Au*/*porous Ti_3_C_2_T_*x*_-TiO_2_/Ag	∼10^5^	300	2 × 10^3^ s	0.2 V	3 (Ternary)	*This work*

## 4 Conclusion

We demonstrated a bipolar ternary resistive switching performance in a partially oxidized porous Ti_3_C_2_T_*x*_ nonvolatile memory device. Ternary resistive switching functionality is achieved with a high endurance period of 2000 s and 300 stable switching cycles at low voltage regimes. Also, precise control over SET, RESET, and intermediate state voltages reveals the reliability of our devices while switching among three distinct resistance levels. The ternary resistive switching performance is attributed to the drift of negatively charged oxygen and metal ions. Our material initiated hierarchical nanopores in the Ti_3_C_2_T_*x*_ MXene, which are responsible for highly enhanced semiconductivity properties and ternary switching behavior, paving the way for its application in data storage device technology and neuromorphic computing.
